# The Impact of Chlorine Disinfection of Hospital Wastewater on Clonal Similarity and ESBL-Production in Selected Bacteria of the Family Enterobacteriaceae

**DOI:** 10.3390/ijerph192113868

**Published:** 2022-10-25

**Authors:** Damian Rolbiecki, Ewa Korzeniewska, Małgorzata Czatzkowska, Monika Harnisz

**Affiliations:** Department of Water Protection Engineering and Environmental Microbiology, Faculty of Geoengineering, University of Warmia and Mazury in Olsztyn, Prawocheńskiego 1, 10-720 Olsztyn, Poland

**Keywords:** hospital wastewater, wastewater disinfection, chlorination, Enterobacteriaceae, antibiotic resistance, ESBL, ERIC-PCR

## Abstract

Hospitals are regarded as ecological niches of antibiotic-resistant bacteria (ARB). ARB can spread outside the hospital environment via hospital wastewater (HWW). Therefore, HWW is often disinfected in local stations to minimize that risk. Chlorine-based treatment is the most popular method of HWW disinfection around the world, however, recent research has suggested that it can contribute to the spread of antimicrobial resistance (AMR). The aim of this study is to determine the impact of HWW disinfection on the clonal similarity of Enterobacteriaceae species and their ability to produce extended-spectrum beta-lactamases (ESBLs). The study was conducted in a hospital with a local chlorine-based disinfection station. Samples of wastewater before disinfection and samples of disinfected wastewater, collected in four research seasons, were analyzed. Bacteria potentially belonging to the Enterobacteriaceae family were isolated from HWW. The Enterobacterial Repetitive Intergenic Consensus Polymerase Chain Reaction (ERIC-PCR) method was used to generate DNA fingerprints of all bacterial isolates. The isolates were phenotypically tested for the production of ESBLs. Antibiotic resistance genes (*bla*_SHV_, *bla*_TEM_, and *bla*_OXA_, *bla*_CTX-M-1-group_, *bla*_CTX-M-2-group_, *bla*_CTX-9-group_ and *bla*_CTX-M-8/25-group_) were detected by PCR in strains with confirmed phenotypic ability to produce ESBLs. The ESBL+ isolates were identified by the sequencing of 16S rDNA. In the present study, the same bacterial clones were isolated from HWW before and after disinfection and HWW was sampled in different seasons. Genetic and phenotypic variations were observed in bacterial clones. ESBL+ strains were isolated significantly more often from disinfected than from non-disinfected HWW. The *bla*_OXA_ gene was significantly more prevalent in isolates from disinfected than non-disinfected HWW. *Enterobacter hormaechei* and *Klebsiella pneumoniae* were the dominant species in ESBL+ strains isolated from both sampling sites. The results of this study indicate that chlorine-based disinfection promotes the survival of ESBL-producing bacteria and/or the transmission of genetic determinants of antimicrobial resistance. As a result, chlorination increases the proportion of ESBL-producing Enterobacteriaceae in disinfected wastewater. Consequently, chlorine-based disinfection practices may pose a risk to the environment and public health by accelerating the spread of antimicrobial resistance.

## 1. Introduction

Antimicrobial resistance (AMR) poses one of the greatest challenges in contemporary medicine. Hospitals are regarded as ecological niches of antibiotic-resistant bacteria (ARB). Prolonged hospitalization and the widespread use of antibiotics contributes to ARB selection and the spread of highly drug-resistant strains in the hospital environment [1]. ARB can cause various infections in the hospital setting, including local hospital epidemics [2]. Drug-resistant pathogenic microorganisms can also spread outside the hospital environment. Hospital wastewater (HWW) is one of the main carriers of highly pathogenic and multidrug-resistant microorganisms. There is evidence to indicate that drug-resistant strains colonizing patients can spread outside the hospital environment via HWW [3,4]. Therefore, HWW is often disinfected in local stations to minimize that risk. Pathogenic bacteria colonizing HWW are inactivated during the disinfection process in order to prevent the spread of dangerous pathogens to municipal sewage systems. This stage is very important because pathogenic bacteria can reach municipal wastewater treatment plants (WWTPs), where they can survive biological treatment and eventually reach the natural environment with effluents. Wastewater can be disinfected with the use of chemical and physical methods. Chemical disinfection involves chlorine, and it is widely used around the world due to its broad spectrum of antibacterial activity, high effectiveness, and low cost [5].

Chlorine is an oxidizing agent that reacts non-selectively with cell components and affects metabolic processes. It has been suggested that the cytoplasmic membrane is a possible key target involved in bacterial inactivation by chlorine [6]. However, chlorine can also damage nucleic acids and affect enzyme activity [7]. Bacteria can survive high chlorine concentrations, such as 2.0 mg/L free chlorine for 1 h [8]. Chlorine-injured bacteria can regrow, and their biological activity can be restored [9]. Chlorine-based disinfection eliminates microorganisms, but it can also contribute to the spread of drug resistance. Recent research has shown that chlorination can affect the frequency of horizontal gene transfer (HGT) in bacterial communities [10,11].

Chlorination also generates toxic disinfection byproducts (DBP), the quantity and complexity of which are determined by the concentrations of naturally occurring substances in wastewater. This increases the cytotoxicity and genotoxicity of wastewater organic matter after chlorination. Disinfection byproducts can be toxic to flora and fauna in water bodies that receive wastewater, and they can also exert adverse effects on the microorganisms and plankton that colonize these ecosystems [12,13].

Bacteria of the Enterobacteriaceae family that produce extended-spectrum beta-lactamases (ESBLs) play a very important role in the hospital setting and during AMR monitoring in the natural environment. According to the World Health Organization (WHO) [14], these microorganisms constitute high-priority alert pathogens that require novel therapeutic methods. Recent AMR surveillance reports in the EU focus on *E. coli* and *Klebsiella pneumoniae* as the key representatives of the Enterobacteriaceae family [15]. In this bacterial family, ESBL production mechanisms are determined primarily by Ambler Class A beta-lactamases (SHV, TEM, CTX-M). In the 1990s, TEM- and SHV-type ESBLs were the predominant ESBL types. A total of 253 TEM variants and 247 SHV variants have been described to date [16]. Not all TEM and SHV variants produce ESBLs, and they possess either a narrow (e.g., TEM-1, SHV-1, SHV-11) or a broad spectrum of ESBL activity (e.g., TEM-24, SHV-5, SHV-12) [17,18]. CTX-M-type ESBLs have become increasingly common worldwide in recent decades. These enzymes are the most prevalent ESBLs in Enterobacteriaceae, in Europe and in other parts of the world. All of the described CTX-M variants are potential ESBL producers [18]. A total of 256 CTX-M variants have been described to date [16]. They have been divided into five groups: (I) CTX-M-1 (including CTX-M-1, 15, 68, 32, 3, 28, 55, 57, 71, 117); (II) CTX-M-2 (including CTX-M-2, 31, 43, 131, 20, 124); (III) CTX-M-8 (including CTX-M-8, 40, 63), (IV) CTX-M-9 (including CTX-M-9, 21, 126, 93, 14, 67); (V) CTX-M-25 (including CTX-M-25, 78, 152, 26, 39, 91, 89, 94, 41, 100), and each group is named after the first described member [19]. In Enterobacteriaceae, ESBL mechanisms can be also conditioned by the production of Ambler Class D OXA-type enzymes, which constitute a large and diverse family of beta-lactamases. A total of 1 120 OXA variants have been described to date [16], including narrow-spectrum beta-lactamases (such as OXA-20 and OXA-47), ESBLs (such as OXA-32 and OXA-53), and beta-lactamases that confer resistance to carbapenems (such as OXA-48 and OXA-23).

The aim of this study is to determine the impact of HWW disinfection on the clonal similarity of the Enterobacteriaceae species and their ability to produce ESBLs. The following parameters were analyzed: (I) Enterobacterial Repetitive Intergenic Consensus Polymerase Chain Reaction (ERIC-PCR) fingerprints of isolates, to identify genetic clones capable of surviving disinfection, and to determine phenotypic and genotypic differences between these isolates; (II) changes in the abundance of ESBL+ isolates before and after HWW disinfection; (III) changes in the species composition of ESBL+ isolates, and changes in the genetic resistance mechanisms of ESBLs.

## 2. Materials and Methods

### 2.1. Study Site and Sampling

**Figure 1:**
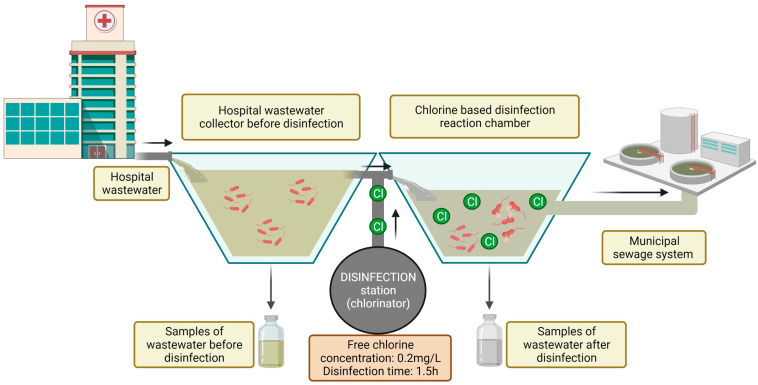
Diagram of the study site and the sampling procedure.

The study was conducted in a hospital in north-eastern Poland. The hospital is located in a suburban area, and it treats patients with respiratory diseases. The hospital has approximately 200 beds in eight wards. Annual patient volume exceeds 11,000. The hospital has an infectious disease ward, and the effluents must be disinfected in line with Polish law [20]. The hospital has a local disinfection station, which is equipped with a dosing station (chlorinator) and dosing pumps. Wastewater is disinfected by an automated electrolytic sodium hypochlorite (NaClO) generation system. The disinfectant is supplied to the reaction chamber based on the volume of incoming wastewater. The dosing station was programmed to obtain a final concentration of free chlorine in the reaction chamber of 0.2 mg/L of wastewater. Wastewater remains in the reaction chamber for 1.5 h. After this time, the effluent is discharged to the municipal sewage system (Figure 1).

Samples of wastewater before disinfection and samples of disinfected wastewater were analyzed. The samples were collected in four seasons: spring (20 April 2021), summer (24 August 2021), fall (16 November 2021), and winter (3 February 2022). The hospital was not equipped with an automatic sewage sampler, enabling the collection of 24-h pooled samples. Therefore, an aggregate sample composed of three grab samples, with an estimated volume of 1 L, was collected. A total of eight wastewater samples were obtained, including four samples from each sampling site. The samples were transported to the laboratory on the day of collection and analyzed.

### 2.2. Isolation of Bacteria from Hospital Wastewater Samples

Wastewater samples were homogenized and diluted. Sample specimens of 0.1 mL of each serial dilution (10^−1^, 10^−2^, 10^−3^, 10^−4^) were inoculated on culture media. Two types of media were used to isolate bacteria potentially belonging to the Enterobacteriaceae family. The following growth media for isolating high priority Enterobacteriaceae species were used: (I) m-FC Agar (Sigma-Aldrich, St. Louis, MO, USA, Merck, Kenilworth, NJ, USA), with the addition of p-rosolic acid solution in 0.2 M Sodium hydroxide solution–to isolate *Escherichia coli*; (II) Klebsiella ChromoSelect Selective Agar (KCSA) (Sigma-Aldrich, Merck), with the addition of Klebsiella Selective Supplement (Sigma-Aldrich, Merck)–to isolate *Klebsiella* spp. The samples were plated on each medium in duplicate and incubated for 24 h at 44.5 °C for m-FC and 37 °C for KCSA.

After incubation, specific bacterial colonies (m-FC-blue colored colonies; KCSA-purple/magenta (mucoid) colonies) were transferred to solid TSA for further analyses. A total of 240 bacterial colonies (120 from m-FC and 120 from KCSA), including 60 colonies from each sampling season (30 colonies plated on m-FC and 30 colonies plated on KCSA), were randomly selected from each sampling site. A total of 480 bacterial isolates were obtained.

### 2.3. Phenotypic Detection of ESBL Producers

Each of the 480 isolates was phenotypically tested for the production of ESBLs. The phenotypic detection was carried out in two stages. In the first step, each isolate was plated on a chromogenic medium (ESBL chromID™, bioMérieux, Warszawa, Poland) to screen for ESBL-producing Enterobacteriaceae, and it was incubated for 24 h at 37 °C. After incubation, 130 strains were cultured on the chromID™ ESBL medium. These strains were regarded as potential ESBL producers (P-ESBL).

In the second step, each of the 130 P-ESBL isolates were subjected to the Combined Disc Test (CD), according to EUCAST guidelines [21]. An inoculum consisting of 0.5 McFarland dilutions of all tested strains was prepared in 3 mL of normal saline with the use of 0.5 McFarland standards. The inoculums were evenly plated on an Mueller Hinton agar, using the lawn streaking method. The plates were left to dry for 5 min. The ESBL Detection Disc Set (Mast Group, Merseyside, UK) was then used to confirm ESBL production. Three paired sets of antibiotic discs were used to confirm the presence of ESBLs: (I) ceftazidime discs (30 µg), ceftazidime (30 µg) + clavulanic acid (10 µg) discs; (II) cefotaxime (30 µg) discs, cefotaxime (30 µg) + clavulanic acid (10 µg) discs; (III) cefpodoxime (10 µg) discs, cefpodoxime (10 µg) + clavulanic acid (1 µg) discs. The plates with antibiogram sets were incubated at 35 ± 2 °C for 17 ± 1 h. After incubation, the zone of inhibition for each antibiotic and antibiotic + clavulanic acid was compared. ESBL production was confirmed when the inhibition zone increase reached ≥ 5 mm in the presence of clavulanic acid for any or all of the sets. Twenty-six P-ESBL strains that had been successfully cultured on the chromID™ ESBL medium had tested negative in the disc test. A total of 104 ESBL+ strains were obtained, including 28 from non-disinfected wastewater and 76 from disinfected wastewater.

In both detection stages, *E. coli* ATCC 25922 and *K. pneumoniae* ATCC 15380 were used as the negative and positive quality control, respectively.

### 2.4. Isolation of Bacterial Genomic DNA

Bacterial genomic DNA was isolated using the heat treatment method [22], with minor modifications [23]. Bacterial strains were suspended in 0.5 mL of double-distilled water (ddH2O). The bacterial suspension was incubated at 95 °C for 10 min in the Grant QBD4 Block Heater (Grant Instruments) and centrifuged for 5 min at 4000× *g* rpm. The supernatant (DNA) was transferred to a sterile Eppendorf tube. The quality and quantity of the obtained genetic material were evaluated with the Multiskan SkyMicroplate Spectrophotometer (Thermo Scientific, Waltham, MA, USA). DNA was stored at −20 °C for further analysis.

### 2.5. ERIC-PCR Fingerprinting

The ERIC-PCR method was used to generate DNA fingerprints of all 480 bacterial isolates. The ERIC-PCR reaction was performed according to the procedure described by Versalovic et al. (1991) [24], with minor modifications [23], using ERIC 1 and ERIC 2 primers (Table S1). PCR products were separated on 1.5% agarose gel (Sigma Aldrich, Merck) stained with ethidium bromide (0.5 mg/mL). Digital image data from electrophoresis gels were obtained using the Gel Doc EZ System and Image Lab™ Software (Bio-Rad Laboratories, CA, USA). Digital images of the gels were used for further data analyses.

### 2.6. Detection of Antibiotic Resistance Genes Using Polymerase Chain Reaction (PCR)

Antibiotic resistance genes were detected by PCR in only 104 strains, with a confirmed phenotypic ability to produce ESBLs. The PCR method was used to detect beta-lactam resistance genes: *bla*_SHV_, *bla*_TEM_, and *bla*_OXA_, and specific groups of CTX-M beta-lactamases (*bla*_CTX-M-1-group_, *bla*_CTX-M-2-group_, *bla*_CTX-9-group_ and *bla*_CTX-M-8/25-group_) [25,26]. PCR was performed in a reaction mix with a volume of 15 μL, containing a pair of specific primers (Table S1), 1 μL of genomic DNA of each sample, and the NZYTaq II 2 × Green Master Mix. PCR products were separated electrophoretically by transferring 5 μL of each amplified DNA fragment to 1.5% agarose gel stained with ethidium bromide (0.5 μg/mL) (Sigma, St. Louis, MO, USA). Electrophoresis was conducted for 1 h at 100 V in 0.5 × TBE buffer. Positive control for individual genes was used in each PCR reaction.

### 2.7. Identification of ESBL+ Isolates

A total of 104 ESBL+ isolates were identified by PCR amplification and sequencing (Genomed S.A., Warsaw, Poland) of 16S rDNA [27]. The obtained sequences were blasted against the reference sequences in GenBank (https://blast.ncbi.nlm.nih.gov/Blast.cgi (accessed on 29 June 2022)) to identify bacterial strains.

### 2.8. Data Analysis

The electrophoretic distribution of ERIC-PCR fingerprints was analyzed with GelJ jv1.0 software [28]. Automatic photo-normalization was applied to compare banding patterns within the same gel and between different gels. The image was analyzed automatically, and manually corrected if needed. A total of 102 distinct amplified loci were detected within ERIC-PCR profiles, and their molecular weight ranged from 141.39 to 5057.87 bp.

Gel images were pre-processed, and ERIC-PCR profiles were analyzed for similarities by calculating the Dice coefficient and Pearson’s correlation coefficient. The unweighted-pair group method with arithmetic mean (UPGMA) was used as the linkage method in both approaches. In the Dice coefficient approach, similarities are assessed based on the presence or absence (0/1 values) of bands of the same molecular weight in fingerprint patterns. Pearson’s correlation coefficient was calculated based on the intensity of ethidium bromide fluorescence along normalized migration distances for the two fingerprints. In both approaches, error tolerance was set at 5% in order to neutralize the electrophoresis running distance, background signal intensity, and random variability in the normalization of gel images, according to the procedure described by Chokesajjawatee et al. (2008) [29]. The results of the analysis were used to generate a matrix of similarities between the isolates and dendrograms.

The similarity matrix based on Pearson’s correlation coefficient was used for further statistical analyses and visualizations, according to the procedure described by Chokesajjawatee et al. (2008) [29]. Dice coefficient values were used to confirm the identification of bacterial clones between the isolates.

According to the methodology proposed by Chokesajjawatee et al. (2008) [29], strains with fingerprint similarity of 90% to 100% were indistinguishable when Pearson’s correlation coefficient was applied to analyze normalized densitometric curves. Pearson’s correlation coefficient of ≥97% was used as a border value to discriminate clones with high accuracy. The strains showing high values of Pearson’s correlation coefficient were further analyzed to ensure that they had the same band numbers and positions. The isolates characterized by Pearson’s correlation coefficient of ≥97% and Dice similarity coefficient of 100% were considered clones.

The similarity matrix based on Pearson’s correlation coefficient was used to perform and visualize non-metric multidimensional scaling (NMDS) in R studio with vegan and ggplot2 packages. Bar and pie charts and heatmaps were generated, and the Chi-square test was performed in GraphPad Prism Software. The network analysis was conducted in Gephi 0.9.6 software using Force Atlas 2 and Noverlap layouts. The Chi-square test was used to detect statistically significant differences between sampling sites (*p* ≤ 0.05). The mean cumulative number of antibiotic resistance genes per isolate (ARGs_index_) was determined by summing up the total number of ARGs in selected groups of bacteria and dividing the result by the number of the analyzed strains within groups.

## 3. Results

**Figure 2:**
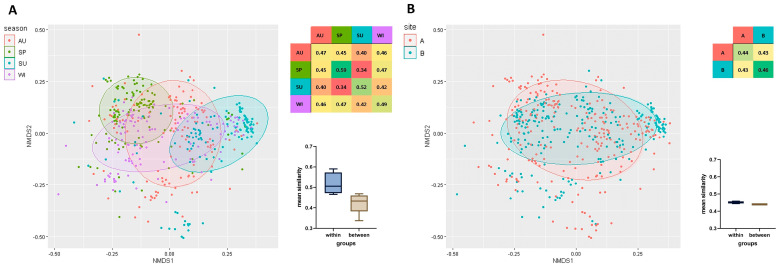
Non-metric multidimensional scaling (NMDS) based on a matrix of clonal similarity of bacterial strains, including a 95% confidence ellipse. Each point represents one bacterial isolate. A heatmap of similarities within and between the studied groups is presented. The barplot (Box-Whisker Plot) of average similarity within and between groups (whiskers denote minimum and maximum values) and the box represent the second and third quartile with the median bar inside. The results are presented for each season (**A**) and sampling site (**B**). Legend: AU—autumn, SP—spring, SU—summer, WI—winter, B—before disinfection, A—after disinfection.

A total of 480 strains isolated from HWW were analyzed, and their clonal similarity was evaluated based on ERIC-PCR fingerprints. The mean clonal similarity of all isolates was determined at 0.44. Genetic similarity was compared within the examined isolate groups, separately for each season (Figure 2A) (irrespective of sampling sites), as well as for each sampling site (Figure 2B) (irrespective of season). Mean clonal similarity was higher within groups (seasons/sites) than between groups. Season exerted a greater influence on the genetic similarity of bacterial strains than disinfection (Figure 2). Strains isolated in spring (SP, 0.59) were characterized by the greatest similarity, followed by the strains isolated in summer (SU, 0.52), winter (WI, 0.49), and autumn (AU, 0.47). Clonal similarity was highest in strains isolated in spring and winter (0.47), and lowest in strains isolated in spring and summer (0.34). Strains isolated from HWW before disinfection were somewhat more similar (B, 0.46) than those isolated after chlorination (A, 0.44). The mean similarity of strains isolated from HWW before and after disinfection reached 0.43 (Figure 2).

**Figure 3:**
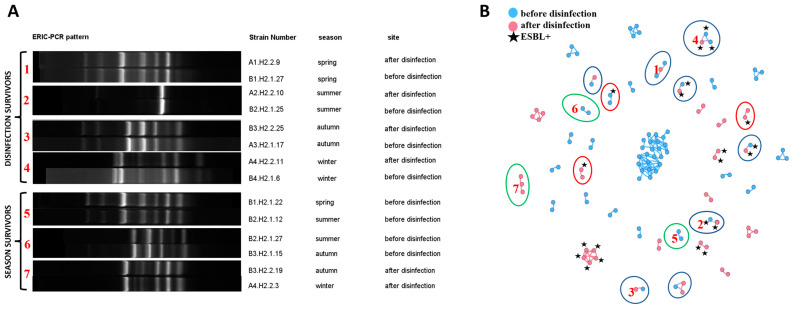
Presentation of clonal relationships: (**A**) Examples of ERIC-PCR fingerprints of isolates regarded as clones: between sampling sites (disinfection survivors) and seasons (season survivors); (**B**) Network analyses showing all clonal relationships within the studied isolates. Blue nodes represent strains isolated from wastewater before disinfection, and pink nodes represent isolates from disinfected wastewater. ESBL+ isolates are marked with a black star. The clones identified in isolates from different sampling sites are marked with a blue ellipse. The clones identified in isolates from different seasons are marked with a green ellipse. Clones that differed in ESBL production are marked with a red ellipse. The connections between nodes represent Pearson’s correlation coefficient of ≥97% and the Dice similarity coefficient of 100% (clones). Nodes = 117; Edges = 133. Red numbers in (**A**) relate to the numbers in (**B**) and denote the same bacterial relationships.

Bacterial clones (strains showing high similarity confirmed by both values of Pearson’s correlation coefficient of ≥97% and the Dice coefficient of 100% in the fingerprint analysis) in the fingerprint analysis) were identified in the ERIC-PCR assay. A total of 133 clonal similarities were identified in the group of 115,200 analyzed relationships (around 0.12%) (Table S2). Bacterial clones were distributed in 40 separate groups, where the largest group was comprised of 24 isolates, and the smallest group of two isolates (Figure 3B). Bacterial clones were significantly more frequently identified in samples collected in the same season and site (85.82% of the cases). The same bacterial clones were isolated from HWW before and after disinfection (11.9%) (disinfection survivors are presented in Figure 3A and are marked with a blue ellipse in Figure 3B). Isolates with identical ERIC-PCR fingerprints were also detected in HWW sampled in different seasons (2.99%) (season survivors are presented in Figure 3A and are marked with a green ellipse in Figure 3B). Bacterial clones isolated from HWW samples before and after disinfection were only identified in the same season. Isolates with the same fingerprints were only detected in two successive seasons (spring-summer, summer-autumn, autumn-winter).

**Figure 4:**
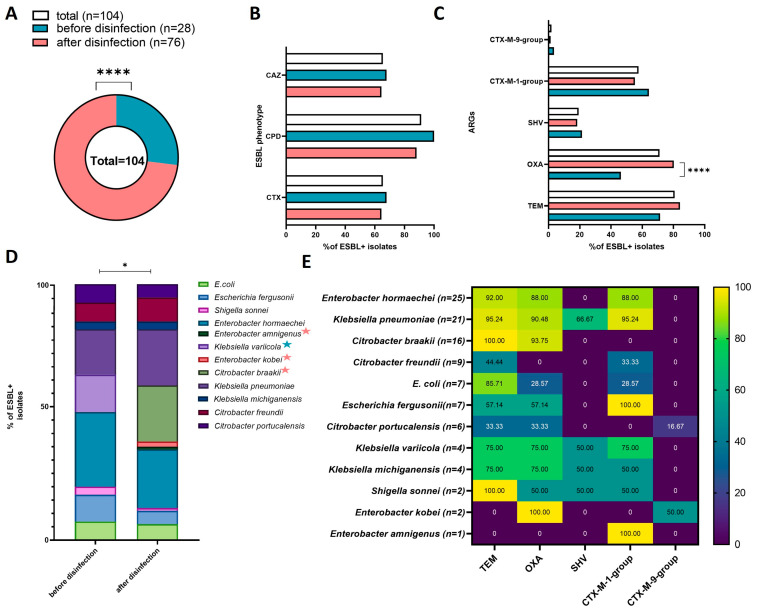
Prevalence and characteristics of ESBL+ isolates in each sampling site and in all sites: (**A**) pie chart presenting the number of ESBL+ isolates; (**B**) bar chart presenting the percentage of ESBL+ isolates with the phenotypic ability to produce ESBL against the tested antibiotics in the Combined Disc Test (cefpodoxime (CPD), ceftazidime (CAZ), cefotaxime (CTX)); (**C**) percentage of ARGs in ESBL+ isolates; (**D**) percentage of bacterial species in ESBL+ isolates from sampling sites (species identified in only one site are marked with a colored star; star colors are explained in the legend of chart A); (**E**) heatmap presenting the percentage of ARGs in the total number of ESBL+ isolates. Black stars denote significant differences at * *p* < 0.05, **** *p* < 0.0001.

Twenty-eight of the strains isolated from HWW before disinfection (*n* = 240) were ESBL producers (11.67%, 28/240). In the group of strains isolated from disinfected HWW (*n* = 240), 76 isolates were capable of producing ESBL (31.67%, 76/240). A total of 104 ESBL+ strains were obtained (21.67%, 104/480). ESBL+ strains were significantly more often isolated from disinfected than from non-disinfected HWW (X^2^ = 28.2815, *p* < 0.00001) (Figure 4A).

The Combined Disc Test analyzing bacteria’s susceptibility to cefpodoxime (CPD), cefotaxime (CTX), and ceftazidime (CAZ), revealed no significant differences in the type of phenotypic activity of ESBL+ strains before and after disinfection (Figure 4B). Most ESBL+ isolates were resistant to CPD (100% of isolates from non-disinfected HWW, 88.6% of isolates from disinfected HWW; 91.35% in total), and the number of isolates resistant to CTX and CAZ were similar (67.86% of isolates from non-disinfected HWW, and 64.47% of isolates from disinfected HWW; 65.38% in total).

The obtained ESBL+ isolates differed in the prevalence of ARGs (Figure 4C). The *bla*_TEM_ gene was most prevalent, and was identified in 84 isolates (80.77%, 84/104), including 20 isolates from non-disinfected HWW (71.43%, 20/28) and 64 isolates from disinfected HWW (84.21%, 64/76). The *bla*_OXA_ gene was identified in 74 strains (71.15% 74/104), and it was significantly more prevalent in isolates from disinfected (80.26%, 61/76) than non-disinfected HWW (46.43%, 13/28) (X^2^ = 11.4123, *p* = 0.00073). The *bla*_CTX-M-1-group_ gene was identified in 60 isolates (57.69% 60/104), including 18 (64.29%, 18/28) and 42 (55.26%, 42/76) isolates from non-disinfected and disinfected HWW, respectively. The *bla*_SHV_ gene was detected in 20 isolates (19.23%, 20/104), including six isolates from non-disinfected HWW (21.43% 6/28) and 12 isolates from disinfected HWW (18.42%,12/76). The *_bla_*_CTX-M-9-group_ was least prevalent, and it was identified in only two isolates (1.92%, 2/104), including one isolate from non-disinfected HWW (3.57%, 1/28) and one isolate from disinfected HWW (1.32%, 1/76). None of the analyzed isolates harbored *bla*_CTX-M-2-group_ or *_bla_*_CTX-M-8/25-group_ genes.

All 104 ESBL+ isolates were identified to species level in the 16S rDNA sequencing analysis. The examined ESBL+ isolates were characterized by high species diversity, despite the fact that culture media targeting *Escherichia coli* and *Klebsiella* spp. were used in the study. Twelve Enterobacteriaceae species were identified: *Enterobacter hormaechei* (*n*= 25), *Klebsiella pneumoniae* (*n* = 21), *Citrobacter braakii* (*n* = 16), *Citrobacter freundii* (*n* = 9), *Escherichia coli* (*n* = 7), *Escherichia fergusonii* (*n* = 7), *Citrobacter portucalensis* (*n* = 6), *Klebsiella variicola* (*n* = 4), *Klebsiella michiganensis* (*n* = 4), *Shigella sonnei* (*n* = 2), *Enterobacter kobei* (*n* = 2), and *Enterobacter amnigenus* (*n* = 1). The species diversity of bacterial strains isolated from HWW before and after disinfection is presented in Figure 4D. Significant differences were observed in the species composition of the tested isolates (X^2^ = 19.98, *p* = 0.0457). *Enterobacter hormaechei* and *Klebsiella pneumoniae* were the dominant species in ESBL+ strains isolated from both sampling sites. *Klebsiella variicola* was detected only in non-disinfected HWW, whereas *Enterobacter amnigenus*, *Enterobacter kobei*, and *Citrobacter braakii* were isolated only from disinfected HWW. The prevalence of the evaluated ARGs differed across the identified bacterial species (Figure 4E). The most prevalent genes were *bla*_TEM_ and *bla*_OXA_, which were identified in 10 species, followed by *bla*_CTX-M-1-group_ (9 species), and *bla*_SHV_ (4 species). The *bla*_CTX-M-9-group_ was detected in only two species: *Citrobacter portucalensis* and *Enterobacter kobei*. *Enterobacter amnigenus* was characterized by the lowest diversity of ARGs.

The co-occurrence of ARG profiles in ESBL+ isolates is presented in Table 1. The *bla*_TEM_ + *bla*_OXA_ + *bla*_CTX-M-1-group_ combination was most prevalent, and it was detected in 30 isolates. *bla*_CTX-M-9-group_, *bla*_SHV_, and the combination of *bla*_OXA_ + *bla*_CTX-M-9-group_ were least frequently detected. Seven ESBL+ isolates did not harbor any of the analyzed ARGs. The co-occurrence of four ARGs (*bla*_TEM_ + *bla*_OXA_ + *bla*_SHV_ + *bla*_CTX-M-1-group_) was noted only in *Klebsiella* spp.

**Figure 5:**
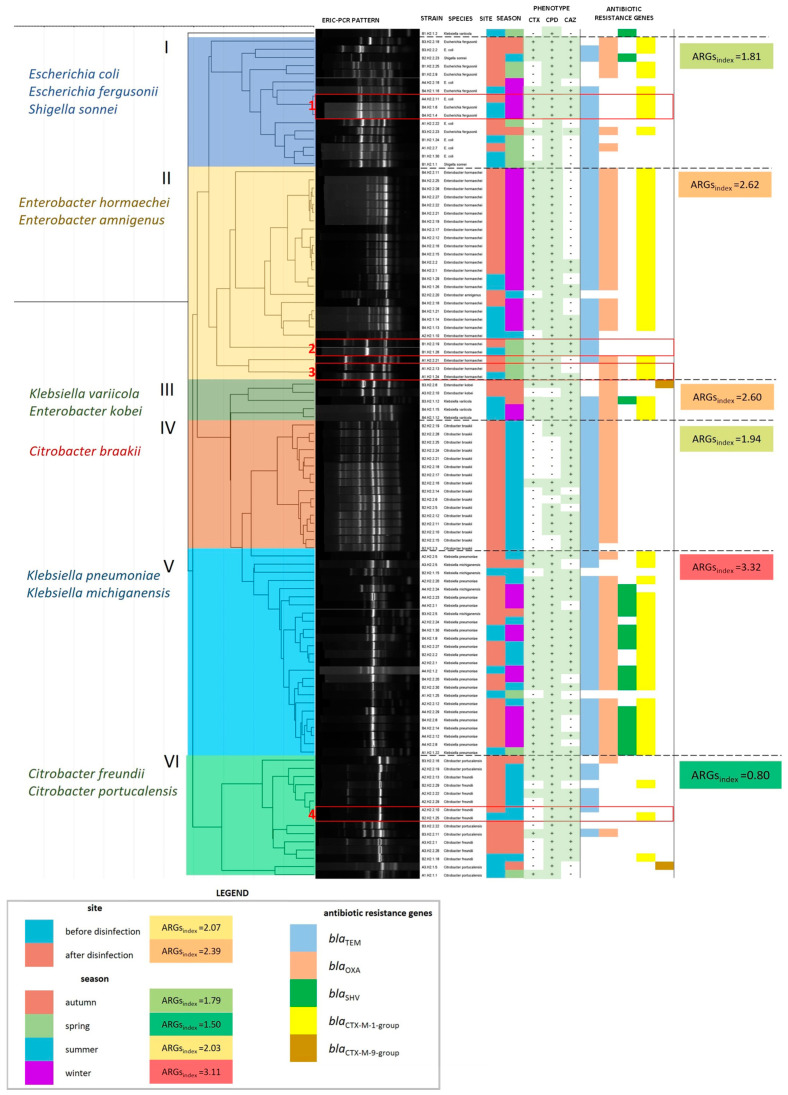
A dendrogram based on the similarity matrix of ERIC-PCR patterns in 104 ESBL+ isolates (similarity method: Pearson’s correlation coefficient; linkage method: UPGMA). The dendrogram was divided into six clusters with different species composition (cluster numbers and species names are presented on the left side of the dendrogram). Strain characteristics are presented on the right side of the dendrogram: isolate identifier, species, site and season of isolation, phenotypic characteristics of ESBLs, and the presence of ARGs. The ARG_index_ was calculated for each cluster, season, and sampling site as the mean number of ARGs per bacterial isolate in each group. ESBL+ isolates from different sampling sites, characterized by identical ERIC-PCR profiles, are presented in subgroups in red boxes and are numbered from 1 to 4.

The detailed characteristics of each ESBL+ isolate are presented graphically in Figure 5. The examined isolates were grouped into six main clusters (marked with Roman numerals I–VI in Figure 5) based on the results of the clonal similarity analysis. Each cluster differed in species composition, which is described on the left-side of the dendrogram. The identified groups also differed in ARG_index_ values (mean number of ARGs per 1 bacterial isolate in a cluster). The ARG_index_ value was highest in cluster V (*Klebsiella* spp.) and lowest in cluster VI (*Citrobacter freundii* + *Citrobacter portucalensis*). ARG_index_ was also calculated for groups of isolates from various sampling sites and seasons. This parameter was higher in isolates from disinfected (2.39) than non-disinfected HWW (2.07). ESBL+ strains which were isolated from various sites, but were characterized by identical ERIC-PCR profiles, were divided into four subgroups (marked with Arabic numerals 1–4 in Figure 5). Both the absence and presence of differences in phenotypic drug resistance and ARG profiles were observed in subgroups of ESBL+ genetic clones. No such differences were observed between the clones in subgroups 1 and 2. The clones in subgroup 3 differed in phenotypic drug resistance, but had the same ARG profile. The strains in subgroup 4 differed in both phenotypic drug resistance and ARG profile.

**Figure 6:**
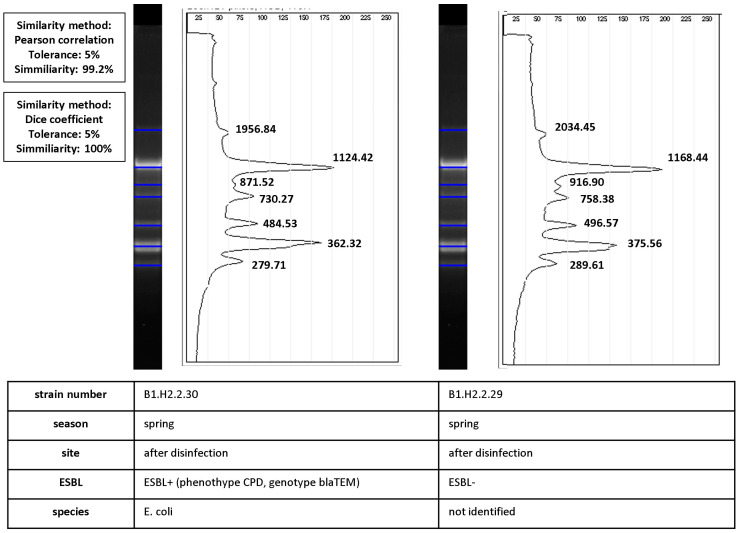
Calculation of similarity values between pairs of ERIC-PCR fingerprints of ESBL+ and ESBL− isolates (isolate characteristics are presented in the table under the diagrams). Band positions were matched by automated band calling in GelJ 1.0. The Dice coefficient (100%) was calculated for match/mismatch data. The line graph shows the intensity of ethidium bromide fluorescence along normalized migration distances for the two fingerprints. Pearson’s correlation coefficient (99.2%) was calculated from intensity values. The black line is the densitometric curve for fingerprints with product band size values (bp).

Bacterial clones also differed in their ability to produce ESBLs. Three such cases were identified during the study, including one before disinfection and two after disinfection (marked with a red ellipse in Figure 3B). Isolates with very high values of similarity coefficients (Dice coefficient—100%, Pearson’s correlation coefficient—99.2%) differed in their phenotypic ability to produce ESBLs. The process of calculating similarities between ESBL− and ESBL+ isolates is presented in Figure 6.

## 4. Discussion

The ERIC-PCR, one of the repetitive sequence-based PCR (rep-PCR) methods, was used in this study. These methods are widely applied in microbiological research, including in analyses of environmental samples [30], to identify the sources of epidemic outbreaks in hospitals [31,32] and in other molecular epidemiology analyses [33]. ERIC-PCR is most often used to differentiate clones in small microbial communities belonging to the same genus or species [34,35]. In the present study, ERIC-PCR was applied to compare a larger group of microorganisms isolated from HWW and potentially belonging to the Enterobacteriaceae family. A total of 480 bacterial strains were fingerprinted in order to identify clonal similarities. The results suggest that chlorination had no significant influence on the clonal similarity of strains colonizing HWW. The clonal similarity of the isolated bacterial strains was modulated to a greater extent by the sampling season. In the studied hospital, the annual patient volume is estimated at 11,000. The admitted patients carry various bacterial strains, which can increase the diversity of microbial communities colonizing HWW throughout the year. The distribution of bacterial species in clusters in the dendrogram (Figure 5) and the results of other studies [36] suggest that ERIC-PCR is an effective method for grouping bacterial isolates based on species and clonal similarity values.

In studies analyzing a large number of bacterial strains from various sources and seasons, the isolates should be reliably identified and differentiated to determine their sources and to monitor the clonal spread of drug-resistant and pathogenic bacteria. The environmental reservoirs of ARB have to be identified in order to effectively monitor their transmission in a given population or environment [37]. In the present study, the clonal similarity analysis supported the identification of genetically related strains (bacterial clones) colonizing HWW before and after disinfection. In a study of *Aeromonas* spp. strains isolated from wastewater before and after chlorination, Mann et al. (2019) [38] grouped the isolates into a single cluster based on their ERIC-PCR fingerprints. These findings indicate that certain clonal cell lines can survive the disinfection process. Various mechanisms underlying resistance to chlorine compounds have been identified in bacterial strains, including changes in membrane permeability, increased expression of efflux pumps, overproduction of extracellular substances or capsule materials, biofilm production, changes in metabolism, or integration with protozoa. However, many of these mechanisms remain unknown [8]. In the present study, clones were detected only in HWW samples collected in two successive seasons (spring-summer; summer-autumn; autumn-winter). In addition, disinfection survivors were isolated only from HWW samples collected in the same season. This could imply that most strains do not survive in HWW for long periods of time and are gradually replaced by new microbial communities that are supplied with the evacuated HWW. Levican et al. (2016) [39] observed a similar trend when analyzing the clonal similarity of *Arcobacters* spp. isolates from municipal wastewater. They found that *Arcobacters* spp. were effectively eliminated during wastewater treatment, and bacterial clones were rarely (0.9% of the isolates) identified in different sampling sites.

The results of the current study indicate that bacteria with identical fingerprints differed in phenotypic drug resistance and ARG profiles. Bacterial clones also differed in their ability to produce ESBLs. Bacterial clones characterized by phenotypic and genetic differences have been identified by the rep-PCR method in previous studies. *Escherichia coli* clones were more resistant to drugs in the presence of zinc and heavy metals [40,41]. Different serotypes were identified in *Yersinia enterocolitica* [42] and *E. coli* [43] clones. Differences in drug-resistance and integron profiles were reported in *E. coli* clones [44,45], whereas *Klebsiella pneumoniae* clones differed in virulence [46]. Changes in the bacterial genome, such as plasmid cloning, do not induce changes in the strain’s fingerprint. Even high numbers of small or large plasmids do not affect the rep-PCR profile [47]. Therefore, wastewater-borne bacteria can acquire new properties (such as drug resistance) via HGT, without any changes in their fingerprints. This phenomenon has been well documented in the present study, where numerous phenotypic and genotypic differences were observed within isolates sharing identical ERIC-PCR profiles. One such case is closely demonstrated in Figure 6. No differences in the fingerprint were indicated despite the dissimilarity in genetic resistance determinants (*bla*_TEM_). These observations highlight the importance of HWW as an environment that promotes the exchange of genetic information between bacteria. The above report suggests that bacterial clones identified by rep-REC should not be regarded as isolates with identical phenotypic and genetic traits. In some cases, isolates with identical rep-PCR profiles are rejected to eliminate genetically homogeneous strains from the analysis [48]. These strains should be regarded as closely genetically related isolates and/or clones originating from the same bacterial cell. Therefore, rep-PCR methods can be reliably used to determine the structure of bacterial communities in epidemiological research, in studies evaluating microbial ecology and evolution, population dynamics, strain survival, and host-microorganism interactions [49].

In this study, bacterial strains were cultured by the lawn streaking method. In this approach, samples are applied directly to the agar plate to promote the growth of target microorganisms, and surplus clones are not artificially generated. Therefore, if more than one isolate belonging to the same clone is obtained, underlying clonal redundancy, i.e., the abundance of a clonal cell line in the sample, is possible [29]. For this reason, we did not interfere with the number of bacterial strains considered in the analysis of ESBL producers before and after disinfection. The same number of isolates from each sampling site were examined, and ESBL+ strains were identified significantly more often in samples of disinfected than non-disinfected HWW (*p* < 0.00001). These results point to an increase in the proportion of drug-resistant bacteria in the total bacteria present in HWW, capable of surviving the chlorination process. The strains isolated from disinfected HWW were also characterized by higher values of ARGs_index_. According to Xiao et al. (2022) [50], chlorine-tolerant strains are also significantly more resistant to antibiotics. There are two possible explanations for the above. Firstly, bacteria resistant to both chlorine and antibiotics could be co-selected during disinfection, because both mechanisms are often encoded together on genetic elements. In addition, the expression of multidrug efflux pumps is the main process that mediates cross-resistance to chlorine and antibiotics. Research has demonstrated that bacterial efflux pumps are over-expressed under exposure to chlorine, which promotes resistance to various antibiotics, including β-lactams [50,51]. Secondly, HGT is enhanced in the presence of chlorine. Bacteria that comes into contact with chlorine compounds possess special properties. It is believed that chlorine-injured bacteria develop a larger number of pili and pores, and their membranes are more permeable. Such bacteria become physiologically competent in HGT processes. Competent bacteria can account for up to 90% of all bacteria after disinfection [10]. These bacteria can be also characterized by a higher frequency of conjugal transfer (2–5 times higher) [52] and transformation (>550 times higher) [10] processes than bacteria not exposed to chlorination. The number of transformation events can also increase in the presence of high concentrations of extracellular DNA (including biologically active antibiotic-resistance plasmids) in the wastewater environment, resulting from the disintegration of chlorine-sensitive cells. In the present study, the abundance of the *bla*_OXA_ gene was significantly higher (*p* = 0.00073) in strains isolated from disinfected HWW, which could suggest that this gene is particularly mobile in HWW or that it co-occurs with chlorine-resistance mechanisms.

In a study conducted in local hospitals, up to 37.1% of *E. coli* strains isolated from HWW were ESBL producers [53]. In the current study, 21.67% of all isolated strains were capable of producing ESBL. A similar percentage of ESBL-producing Enterobacteriaceae (ESBL-E, 24.81%) was reported globally in wastewater [54]. However, the results noted in the present study differ from the mean ESBL-E counts reported by Zaatout et al. (2021) [54] in HWW (33.98%), and they are closer to the values noted in disinfected wastewater (31.67%).

The percentage of Ambler Class A ESBLs (TEM, CTX-M, SHV) did not differ significantly before and after chlorination. These ESBL variants were observed in 80.77%, 59.62%, and 19.23% of all ESBL+ isolates, respectively. Similar values were reported by Tekiner and Özpınar (2016) [55], who identified *bla*_TEM_, *bla*_CTX-M_, and *bla*_SHV_ in 96.4%, 53.7%, and 34.5% of ESBL-E in animals and food samples, respectively. Chagas et al. (2011) [56] also found that *bla*_TEM_ was the most prevalent gene in ESBL-E isolated from HWW in Brazil. The results of the present study confirm that CTX-M-1-group and CTX-M-9-group ESBLs are the most widespread CTX-M variants in Poland and Europe [53,57]. However, the observations that CTX-M is the most common variant of ESBL-E ([57,58], and that *bla*_CTX-M_ (66.82%), *bla*_TEM_ (51.01%), and *bla*_SHV_ (24.59%) are most frequently isolated from wastewater-borne ESBL-E [54], were not confirmed in the present work. 

The results of the current study revealed certain differences in ARG abundance across bacterial species. The *bla*_SHV_ gene was identified mostly in *Klebsiella* spp. (excluding one *Shigella sonnei* isolate). Mutuku et al. (2022) [58] analyzed 87 ESBL+ isolates from HWW and detected *bla*_SHV_ only in *Klebsiella* spp. According to Turner et al. (2009) [59], SHV ESBLs are chiefly responsible for ESBL+ positive phenotypes in *Klebsiella pneumoniae*, most of which are plasmid-encoded.

The *bla*_OXA_ gene encoding Ambler Class D beta-lactamases was identified in 71.15% of ESBL+ isolates. Osińska et at. (2019) [60] reported considerable variations in *bla*_OXA_ concentrations in municipal wastewater. Amador et al. (2015) [61] detected *bla*_OXA_ in 27.8% of ESBL-E, and Liedhegner et al. (2022) [62], in 86% of cefotaxime-resistant *E. coli* isolated from HWW. In the present study, *bla*_OXA_ was the only gene whose abundance differed significantly between HWW samples collected from different sites. In contrast, Xiao et al. (2022) [50] did not report significant correlations between six chlorine-resistance genes and *bla*_OXA_. This result gives particular cause for concern because *bla*_OXA_ is the only carbapenemase gene in the group of the examined beta-lactamases [18]. In the present study, 77.88% of the analyzed isolates harbored more than one beta-lactamase gene. This result is similar to the value noted by Chagas et al. (2011) [56] (75.26%), and higher than the value reported by Korzeniewska et al. (2013) [53] (38%), in studies of ESBL-E in HWW.

According to Khleifat et al. (2006) [63], the species composition of a sample influences the effectiveness of chlorination. In this study, the species composition of ESBL+ isolates in HWW differed before and after disinfection. Some species were present only in samples collected from a single site, which could suggest that chlorination affects the species composition of microorganisms, or that some species are more tolerant to chlorine. Despite these variations, bacterial isolates obtained from different sampling sites did not differ significantly in clonal similarity. Metagenomic studies have demonstrated that disinfection induces changes in the species diversity and richness of microbial communities, but these changes can be reversed over time [64]. In the present study, the species composition of bacteria isolated from HWW was similar to that reported by other authors [58,65,66,67]. Bacteria of the genera *Klebsiella*, *Enterobacter*, *Citrobacter*, and *Escherichia* were predominant in all cases, which could suggest that these genera are the dominant members of the Enterobacteriaceae family in HWW. In the present study, special attention was paid to *Klebsiella* spp., which were characterized by the highest AMR potential. The highest value of ARGs_index_ was noted in the cluster grouping of all *Klebsiella pneumoniae* and *Klebsiella michiganensis* isolates. *Klebsiella* spp. Were also the only isolates containing four of the examined ARGs. Similar results were reported by Fadare et al. (2021) [65], who studied Enterobacteriaceae strains isolated from HWW and found that *Klebsiella* spp. were characterized by the highest phenotypic drug resistance and the highest diversity of ARGs. In the current study, bacteria of the genus *Klebsiella* accounted for 27.88% of all isolated ESBL-E. In the work of Prado et al. (2008) [68], *Klebsiella pneumoniae* accounted for up to 33.3% of all ESBL+ isolates in wastewater evacuated from a hospital WWTP. Our results also support the observations made by Herraiz-Carboné et al. (2021) [69], who concluded that the fate of *Klebsiella pneumoniae* in HWW should be monitored because this bacterial species poses the greatest threat to public health.

In the first WHO list of ARB [14], Enterobacteriaceae resistant to third-generation cephalosporins (ESBL producers) were placed in the critical priority category as pathogens for which new treatments are urgently needed. According to research, *Klebsiella* spp., in particular *Klebsiella pneumoniae*, are the main promoters of antibiotic resistance in the Enterobacteriaceae family. *Klebsiella pneumoniae* and selected determinants of drug resistance (including *bla*_CTX-M_ and *bla*_TEM_) were proposed as effective indicators of AMR dissemination in the environment [70]. According to an ECDC report [71], Poland is the European country with the highest detection rate of multidrug-resistant *Klebsiella pneumoniae* in clinical isolates (percentage of isolates). In Polish hospitals, 51.5% of clinical isolates of *Klebsiella pneumoniae* were resistant to all of the following antimicrobials: fluoroquinolones, third-generation cephalosporins, and aminoglycosides. Resistance to third-generation cephalosporins (associated with ESBLs) was observed in 64.6% of clinical isolates of *K. pneumoniae* (third place in Europe). In this study, similar trends were noted outside the hospital environment, which gives serious cause for concern.

## 5. Conclusions

The results of this study indicate that chlorine-based disinfection promotes the survival of ESBL-producing bacteria and/or the transmission of genetic determinants of AMR. As a result, chlorination increases the proportion of ESBL-producing Enterobacteriaceae in disinfected wastewater. The study also demonstrates that chlorination in hospital disinfection stations is not fully effective and that bacteria of the Enterobacteriaceae family, including ESBL-E, can survive the disinfection process and reach municipal WWTPs with HWW. According to these results, *Klebsiella* spp., and in particular *Klebsiella pneumoniae*, can be the main promoters of antibiotic resistance in the Enterobacteriaceae family. Analyses of clonal similarity suggests that high levels of genetic and phenotypic variation can be observed in bacterial clones isolated from disinfected HWW. These results confirm previous reports, indicating that chlorination contributes to the spread of drug resistance. This observation is particularly worrying because the number of hospitals operating infectious disease departments and disinfecting hospital wastewater increased during the COVID-19 pandemic.

## Figures and Tables

**Table 1:** Co-occurrence of ARG profiles in ESBL+ bacterial isolates.

ARG Profile	Frequency % (*n*)	Carriers
*bla*_TEM_ + *bla*_OXA_ + *bla*_CTX-M-1-group_	28.85% (30)	*Enterobacter hormaechei* (*n* = 20) *Klebsiella pneumoniae* (*n* = 5) *Klebsiella variicola* (*n* = 3) *Escherichia fergusonii* (*n* = 1) *Klebsiella michiganensis* (*n* = 1)
*bla*_TEM_ + *bla*_OXA_	16.35% (17)	*Citrobacter braakii* (*n* = 15) *Citrobacter portucalensis* (*n* = 1) *E. coli* (*n* = 1)
*bla*_TEM_ + *bla*_OXA_ + *bla*_SHV_ + *bla*_CTX-M-1-group_	15.38% (16)	*Klebsiella pneumoniae* (*n* = 14) *Klebsiella variicola* (*n* = 2)
*bla*_TEM_	12.50% (13)	*Citrobacter freundii* (*n* = 4) *Enterobacter hormaechei* (*n* = 3) *E. coli* (*n* = 3) *Citrobacter braakii* (*n* = 1) *Citrobacter portucalensis* (*n* = 1) *Shigella sonnei* (*n* = 1)
none	6.73% (7)	*Citrobacter portucalensis* (*n* = 2) *Citrobacter freundii* (*n* = 2) *E. coli* (*n* = 1) *Klebsiella michiganensis* (*n* = 1) *Klebsiella pneumoniae* (*n* = 1)
*bla*_TEM_ + *bla*_CTX-M-1-group_	4.81% (5)	*Escherichia fergusonii* (*n* = 3) *E. coli* (*n* = 1) *Klebsiella pneumoniae* (*n* = 1)
*bla*_OXA_ *+ bla*_CTX-M-1-group_	4.81% (5)	*Escherichia fergusonii* (*n* = 3) *Enterobacter hormaechei* (*n* = 2)
*bla*_CTX-M-1-group_	3.85% (4)	*Citrobacter freundii* (*n* = 3) *Enterobacter amnigenus* (*n* = 1)
*bla*_OXA_	1.92% (2)	*Enterobacter kobei* (*n* = 1) *Citrobacter portucalensis* (*n* = 1)
*bla*_TEM_ *+ bla*_OXA_	1.92% (2)	*Shigella sonnei* (*n* = 1) *Klebsiella michiganensis* (*n* = 1)
*bla*_SHV_	0.96% (1)	*Klebsiella variicola* (*n* = 1)
*bla*_OXA_ *+ bla*_CTX-M-9-group_	0.96% (1)	*Enterobacter kobei* (*n* = 1)
*bla*_CTX-M-9-group_	0.96% (1)	*Citrobacter portucalensis* (*n* = 1)

## Data Availability

Not applicable.

## Supplementary Materials

The following supporting information can be downloaded at: https://www.mdpi.com/article/10.3390/ijerph192113868/s1, Table S1: Polymerase chain reaction (PCR) primers and parameters; Table S2: Table showing clonal relationships between tested isolates.
