# Supplementary material for: The Impact of Chlorine Disinfection of Hospital Wastewater on Clonal Similarity and ESBL-Production in Selected Bacteria of the Family Enterobacteriaceae
Source: Int J Environ Res Public Health. 2022 Oct 25;19(21):13868. doi: 10.3390/ijerph192113868 (PMC9655713; doi:10.3390/ijerph192113868)
Supplement: Supplementary file 1 [file ijerph-19-13868-s001.zip › ijerph-1963730-supplementary-done.pdf]

International Journal of
Environmental Research and Public Health

Table S1. Polymerase chain reaction (PCR) primers and parameters.

Target Gene	Primer Sequence(5′-3′)	Amplicon Size(bp)	PCR Annealing Temp (◦C)	References
ERIC	ERIC 1<br>ATGTAAGCTCCTGGGGATTCAC<br>ERIC 2<br>AAGTAAGTGACTGGGGTGAGCG	-	52	Versalovic et al. 1991
bla$_{SHV}$,	F<br>GATGAACGCTTTCCCATGATG<br>R<br>CGCTGTTATCGCTCATGGTAA	214	61	Kim et al. 2009
bla$_{TEM}$	F<br>AGTGCTGCCATAACCATGAGTG<br>R<br>CTGACTCCCC GTCGTGTAGATA	431	61	Kim et al. 2009
bla$_{OXA}$	F<br>ATTATCTACAGCAGCGCCAGTG<br>R<br>TGCATCCACGTCTTTGGTG	296	61	Kim et al. 2009
bla$_{CTX\text{-}M\text{-}1\text{-}group}$	F<br>TTAGGAArTGTGCCGCTGyA<br>R<br>CGATATCGTTGGTGGTrCCAT	688	60	Dallenne et al. 2010
bla$_{CTX\text{-}M\text{-}2\text{-}group}$	F<br>CGTTAACGGCACGATGAC<br>R<br>CGATATCGTTGGTGGTrCCAT	404	60	Dallenne et al. 2010
bla$_{CTX\text{-}9\text{-}group}$	F<br>TCAAGCCTGCCGATCTGGT<br>R<br>TGATTCTCGCCGCTGAAG	561	60	Dallenne et al. 2010
bla$_{CTX\text{-}M\text{-}8/25\text{-}group}$	F<br>AACrCrCAGACGCTCTAC<br>R<br>TCGAGCCGGAAsGTGTyAT	326	60	Dallenne et al. 2010

Table S2. Table showing clonal relationships between test isolates.

Clones		Similarity [%]	
Strain number	Strain number	Dice	Pearson
A2.H2.1.13	A2.H2.1.14	100	100
A2.H2.1.5	A2.H2.1.6	100	100
B2.H2.1.9	B2.H2.1.10	100	100
B2.H2.1.28	B2.H2.1.30	100	100
B2.H2.1.23	B2.H2.1.24	100	100
B2.H2.1.22	B2.H2.1.23	100	100
B2.H2.1.8	B2.H2.1.22	100	100
B2.H2.1.22	B2.H2.1.24	100	100
B2.H2.1.26	B2.H2.1.28	100	100
B2.H2.1.7	B2.H2.1.20	100	100
B1.H2.2.5	B1.H2.2.6	100	99
A2.H2.1.23	B2.H2.1.1	100	99
B2.H2.1.8	B2.H2.1.20	100	99

B4.H2.2.21	B4.H2.2.22	100	99
A2.H2.1.21	A2.H2.1.27	100	99
B2.H2.1.20	B2.H2.1.22	100	99
B2.H2.1.16	B2.H2.1.17	100	99
B2.H2.1.8	B2.H2.1.23	100	99
B2.H2.1.20	B2.H2.1.24	100	99
A2.H2.1.30	B2.H2.1.2	100	99
B2.H2.1.20	B2.H2.1.23	100	99
B2.H2.1.29	B2.H2.1.30	100	99
B2.H2.1.26	B2.H2.1.30	100	99
A2.H2.1.6	A2.H2.1.7	100	99
B4.H2.2.15	B4.H2.2.16	100	99
B1.H2.1.22	B2.H2.1.12	100	99
A3.H2.1.29	A3.H2.1.30	100	99
A2.H2.1.4	B2.H2.1.16	100	99
B2.H2.1.8	B2.H2.1.24	100	99
B1.H2.1.30	B1.H2.1.29	100	92
B1.H2.1.21	B1.H2.1.23	100	99
A3.H2.1.24	A3.H2.1.25	100	99
A2.H2.1.6	A2.H2.1.13	100	99
A2.H2.1.5	A2.H2.1.7	100	99
B1.H2.1.16	B1.H2.1.17	100	99
A4.H2.2.3	A4.H2.2.5	100	99
B2.H2.1.9	B2.H2.1.23	100	99
B4.H2.2.1	B4.H2.2.15	100	99
A2.H2.1.13	B2.H2.1.3	100	99
A2.H2.1.5	A2.H2.1.13	100	99
B1.H2.2.7	B1.H2.2.8	100	99
A2.H2.1.6	A2.H2.1.14	100	99
A2.H2.1.5	A2.H2.1.14	100	99
A2.H2.1.26	A2.H2.1.28	100	99
B4.H2.2.1	B4.H2.2.2	100	99
A1.H2.2.13	A1.H2.1.24	100	99
B2.H2.1.10	B2.H2.1.23	100	99
A4.H2.2.7	A4.H2.2.16	100	99
A1.H2.1.12	A1.H2.1.7	100	99
B2.H2.1.7	B2.H2.1.8	100	99
B4.H2.2.1	B4.H2.2.16	100	99
B4.H2.2.12	B4.H2.2.15	100	99
B4.H2.2.2	B4.H2.2.15	100	99
A4.H2.2.21	A4.H2.2.22	100	99
B2.H2.1.28	B2.H2.1.29	100	99
B2.H2.1.9	B2.H2.1.22	100	99
A2.H2.1.4	A2.H2.1.5	100	99
A3.H2.1.22	A3.H2.1.26	100	99
B2.H2.1.7	B2.H2.1.23	100	99
B1.H2.2.8	B1.H2.2.15	100	99
B2.H2.1.7	B2.H2.1.22	100	99
A2.H2.1.23	A2.H2.1.25	100	99
A4.H2.2.11	B4.H2.1.6	100	99
A2.H2.1.7	A2.H2.1.13	100	99
A2.H2.1.25	B2.H2.1.1	100	99
B3.H2.2.3	B3.H2.2.17	100	99
A2.H2.1.7	A2.H2.1.9	100	99

B2.H2.1.7	B2.H2.1.24	100	99
A2.H2.2.10	B2.H2.1.25	100	98
A1.H2.2.9	B1.H2.1.27	100	98
B2.H2.1.9	B2.H2.1.24	100	98
A2.H2.1.26	B2.H2.1.3	100	98
A2.H2.1.4	A2.H2.1.6	100	98
B3.H2.2.25	A3.H2.1.17	100	98
A2.H2.1.4	B2.H2.1.17	100	98
A2.H2.1.7	A2.H2.1.14	100	98
B2.H2.1.10	B2.H2.1.22	100	98
B2.H2.1.8	B2.H2.1.9	100	98
B4.H2.2.2	B4.H2.2.16	100	98
B2.H2.1.10	B2.H2.1.24	100	98
B3.H2.1.25	B3.H2.1.26	100	98
B3.H2.1.29	B3.H2.1.30	100	98
B2.H2.1.8	B2.H2.1.10	100	98
B1.H2.2.7	B1.H2.2.15	100	98
A2.H2.1.24	A2.H2.1.27	100	98
A4.H2.2.18	A4.H2.2.20	100	98
B4.H2.2.1	B4.H2.2.12	100	98
B2.H2.1.17	B2.H2.1.19	100	98
A4.H2.1.23	A4.H2.1.25	100	98
B4.H2.2.2	B4.H2.2.12	100	98
B2.H2.1.26	B2.H2.1.29	100	98
A2.H2.1.6	B2.H2.1.3	100	98
A4.H2.2.13	A4.H2.2.16	100	98
A2.H2.1.4	A2.H2.1.7	100	98
A2.H2.1.5	B2.H2.1.3	100	98
A4.H2.1.9	A4.H2.1.12	100	98
B2.H2.1.19	B2.H2.1.20	100	98
B2.H2.1.9	B2.H2.1.20	100	98
B2.H2.2.15	B2.H2.2.16	100	98
A1.H2.1.11	A1.H2.1.7	100	98
B2.H2.1.7	B2.H2.1.19	100	98
A4.H2.2.7	A4.H2.2.13	100	98
B2.H2.1.27	B3.H2.1.15	100	98
B4.H2.1.4	B4.H2.1.6	100	97
B4.H2.1.28	A4.H2.2.21	100	97
B2.H2.1.10	B2.H2.1.20	100	97
A4.H2.2.11	B4.H2.1.4	100	97
B2.H2.1.9	B2.H2.1.11	100	97
A2.H2.1.7	B2.H2.1.3	100	97
B1.H2.2.3	B1.H2.2.8	100	97
A2.H2.1.2	A2.H2.1.3	100	97
B1.H2.2.19	B1.H2.1.26	100	97
B4.H2.1.28	A4.H2.2.22	100	97
B3.H2.1.8	B3.H2.1.10	100	97
B1.H2.2.3	B1.H2.2.7	100	97
B4.H2.2.12	B4.H2.2.16	100	97
A2.H2.1.3	A2.H2.1.4	100	97
A2.H2.1.5	B2.H2.1.16	100	97
B3.H2.2.19	A4.H2.2.3	100	97
A2.H2.1.13	A2.H2.1.26	100	97
A2.H2.1.21	A2.H2.1.24	100	97

A1.H2.1.12	A1.H2.1.11	100	97
B2.H2.1.7	B2.H2.1.9	100	97
B1.H2.1.28	A1.H2.2.9	100	97
B1.H2.2.21	B1.H2.2.28	100	97
B2.H2.1.7	B2.H2.1.10	100	97
A1.H2.2.18	A1.H2.2.22	100	97
B2.H2.1.8	B2.H2.1.19	100	97
A3.H2.2.18	A3.H2.2.27	100	97
A3.H2.1.11	A3.H2.2.23	100	97
A3.H2.2.9	B3.H2.2.24	100	97
B2.H2.1.16	B2.H2.1.19	100	97
A2.H2.1.6	A2.H2.1.9	100	97

References:

1. Versalovic, J.; Koeuth, T.; Lupski. J.R. Distribution of repetitive DNA sequences in eubacteria and application to fingerprinting of bacterial genomes. Nucleic Acids Res. 1991, 19, 6823-6831.
2. Kim, J.; Jeon, S.; Rhie, H.; Lee, B.; Park, M.; Lee, H.; Lee, J.; Kim, S. Rapid detection of extended spectrum β-lactamase (ESBL) for enterobacteriaceae by use of a multiplex PCR-based method. Infect. Chemother. 2009, 41, 181-184, 10.3947/ic.2009.41.3.181
3. Dallenne, C.; Da Costa, A.; Decré, D.; Favier, C.; Arlet, G. Development of a set of multiplex PCR assays for the detection of genes encoding important β-lactamases in Enterobacteriaceae, J Antimicrob Chemother 2010, 65, 490–495, https://doi.org/10.1093/jac/dkp498
